# Lactic acid bacteria: reviewing the potential of a promising delivery live vector for biomedical purposes

**DOI:** 10.1186/s12934-015-0313-6

**Published:** 2015-09-16

**Authors:** Olivia Cano-Garrido, Joaquin Seras-Franzoso, Elena Garcia-Fruitós

**Affiliations:** Institut de Biotecnologia i de Biomedicina, Universitat Autònoma de Barcelona, Bellaterra, 08193 Cerdanyola del Vallès, Spain; Departament de Genètica i de Microbiologia, Universitat Autònoma de Barcelona, Bellaterra, 08193 Cerdanyola del Vallès, Spain; CIBER de Bioingeniería, Biomateriales y Nanomedicina (CIBER-BBN), Bellaterra, 08193 Cerdanyola del Vallès, Spain; Department of Ruminant Production, Institut de Recerca i Tecnologia Agroalimentàries (IRTA), Torre Marimon, Caldes de Montbui, 08140 Barcelona, Spain

**Keywords:** Lactic acid bacteria, Delivery vector, QPS, Mucosal, Therapy, Treatment

## Abstract

Lactic acid bacteria (LAB) have a long history of safe exploitation by humans, being used for centuries in food production and preservation and as probiotic agents to promote human health. Interestingly, some species of these Gram-positive bacteria, which are generally recognized as safe organisms by the US Food and Drug Administration (FDA), are able to survive through the gastrointestinal tract (GIT), being capable to reach and colonize the intestine, where they play an important role. Besides, during the last decades, an important effort has been done for the development of tools to use LAB as microbial cell factories for the production of proteins of interest. Given the need to develop effective strategies for the delivery of prophylactic and therapeutic molecules, LAB have appeared as an appealing option for the oral, intranasal and vaginal delivery of such molecules. So far, these genetically modified organisms have been successfully used as vehicles for delivering functional proteins to mucosal tissues in the treatment of many different pathologies including GIT related pathologies, diabetes, cancer and viral infections, among others. Interestingly, the administration of such microorganisms would suppose a significant decrease in the production cost of the treatments agents since being live organisms, such vectors would be able to autonomously amplify and produce and deliver the protein of interest. In this context, this review aims to provide an overview of the use of LAB engineered as a promising alternative as well as a safety delivery platform of recombinant proteins for the treatment of a wide range of diseases.

## Background

Most of the existing strategies for the treatment of diseases are focused on the delivery of naked molecules with a therapeutic activity, from chemically synthesized molecules to recombinant proteins produced in diverse platforms such as bacteria, yeast, insect cells and mammalian cells, among others [1]. However, these treatments require in many cases the use of invasive administration methods such as intravenous or subcutaneous injection of the molecule of interest to reach the targeted region [2]. Moreover, soluble purified proteins and other therapeutic compounds frequently show low stability and/or poor efficiency in the organism forcing repeated administration [2], with the subsequent increase in the amount of needed pharmaceutical and the frequent derived increase in toxicity and cost of the treatment [2]. In the case of recombinant therapeutic proteins produced in microbial hosts, biosafety concerns are raised, mainly due to the possible remnants of pyrogenic or inflammatory contaminants that can trigger undesirable immunogenic responses [3]. Given the need to develop an alternative route for the administration, as well as a safety delivery platform, lactic acid bacteria (LAB) have appeared as an appealing option for the production and delivery of therapeutic molecules and antigens of interest [4]. This heterogeneous group of Gram-positive bacteria, in contrast to Gram-negative bacteria such as *E. coli*, do not contain lipopolysaccarides (LPS) attached to the cell membrane. The absence of such endotoxins avoids the generation of an anaphylactic shock when LAB are administered in humans [3]. In this regard, it should be noted that LAB have a long history of safe use by humans, being used for centuries in food production and preservation [5–7]. In this context, some strains have also a long record in their use as probiotic bacteria producer of metabolites and macromolecules able to maintain and promote human health [5, 8]. Then, LAB have been classified as food grade microorganisms [generally recognized as safe (GRAS) organisms by the US Food and Drug Administration (FDA)] and fulfill criteria of the qualified presumption of safety (QPS) according to the European Food Safety Authority (EFSA). Besides, it is important to stress that an exhaustive work has been done in developing different tools for the recombinant protein production using LAB as cell factories [9]. The development of these tools has made possible the development of LAB able to secrete the protein of interest to the extracellular environment, becoming a key aspect when evaluating the potential of these bacteria for mucosal targeting of therapeutic molecules [4] (Table 1). Alternatively, approaches based on protein display anchored to the bacteria cell wall have also been tested [10], being a system that, even not being as effective as secreted protein in terms of protein expression levels, gives a higher protection to the protein in front of degrading and denaturing agents (Table 1). In consequence, these microorganisms can be used for oral, intranasal or vaginal administration for protein delivery purposes, minimizing any potential side effect associated with the classical parenteral or subcutaneous administration of proteins, simultaneously reducing the dose needed.

**Table 1:** Recombinant proteins produced in LAB for biomedical purposes

LAB	Application	Recombinant protein	Expression vector	Promoter	Protein display	References
*Lactococcus lactis*	IDB	Anti-TNFalpha nanobodies	pTREX-derived	P1 (pH dependent)	Secreted	[22]
*Lactococcus lactis*	IDB	Trefoil Factors (TFF)	pTREX-derived	P1 (pH dependent)	Secreted	[23]
*Lactococcus lactis*	IDB	Low calcium response V (LcrV)	pNZYR-derived	P_Usp45_ (constitutive)	Secreted	[21]
*Lactobacillus gasseri/Lactobacillus casei*	IDB	Superoxide Dismutase (SOD)	pSodA pILKS*sodA*	*sodA* native promoter	–	[39, 40]
*Lactococcus lactis*	IDB	IL-10	Cromosome integrated	P_thyA_ (constitutive)	Secreted	[26]
*Lactococcus lactis*	IDB	IL-27	pT1NX-derived	P1 (pH dependent)	Secreted	[35]
*Lactococcus lactis*	IBD	Murine IL-10	pLB263	P_groESL_ (Inducible)	Secreted	[15]
*Lactococcus lactis/Lactobacillus casei*	IDB/colorectal Cancer	Catalase	pSEC:KatE/pLEM415*mnkat*	P_nisA_ (inducible)/P_ldh_ (constitutive)	Cytoplasmatic	[24, 25]
*Lactococcus lactis*	Type 1 diabetes	Pro Insulin/(GAD)-65/IL-10	pT1NX-derived	P1 (pH dependent)	Secreted	[44]
*Lactococcus lactis*	Type 1 diabetes	HSP65-6P277	pCYT:HSP65-6P277/pHJ: HSP65-6P277	P_nisA_ (inducible)/constitutive	Cytoplasmatic/secreted	[43]
*Lactococcus lactis*	Type 1 diabetes	GAD65 and IA-2	–	–	Secreted	[46]
*Lactococcus lactis*	Diabetes	Single-chain insulin analog, SCI-57	pNZPnisA:uspSCI-57	P_nisA_ (inducible)	Secreted	[41]
*Lactococcus lactis*	Type 2 diabetes	Glucagon like peptide-1 (GLP-1)	pUBGLP-1	P1 (pH dependent)	Secreted	[42]
*Lactococcus lactis*	Cancer	HPV-16 E7 antigen	pLB263	P_groESL_ (inducible)	Secreted	[15]
*Bifidobacterium longum*	Breast cancer	Cytosine Deaminase	pBLES100-S-eCD	–	–	[57]
*Bifidobacterium adolescentis*	Cancer	Endostatin	pBV220-derived	P_R_P_L_ (thermoinducible)	Cytoplasmatic	[58]
*Bifidobacterium breve*	Cancer treatment study tool	Luciferase	pLux MC3	P_help_ (constitutive)	Cytoplasmatic	[59]
*Lactococcus lactis*	Cervical cancer	HPV-16 E7	–	P_nisA_ (Inducible)	Anchored	[61]
*Lactococcus lactis*	Cervical cancer	HPV-16 E7	pMG36e	P32 (constitutive)	Cytoplasmatic	[62]
*Lactococcus lactis/Lactobacillus casei*	Cervical cancer	HPV-16 E7	–	–	Anchored	[63]
*Bacillus subtilis*	Cervical cancer	HPV33 L1	pICHPV33L1-NS/B	Pxylose (inducible)	Intracellular	[74]
*Lactobacillus paracasei*	*Bacillus anthracis* infection	Antibody fragment	pAF100-derived/pAF400-derived/pAF900-derived	P_*apf*_ (constitutive)	Secreted/attached/cell anchored	[85]
*Lactobacillus paracasei*	*Rotavirus* infection	Antibody fragment	pLP501-derived	P_ldh_ (constitutive)	Secreted/cell anchored	[87]
*Bifidubacterium longum*	Enterovirus 71 infection	VP1	pBBADs-VP1	–	–	[109]
*Bifidubacterium longum*	Hepatitis C infection	HCV-NS3 peptide	–	–	Cell anchored	[110]
*Lactococcus lactis*	Staphylococal infection	Staphylocococal nuclease	pLB263	P_groESL_ (inducible)	Secreted	[15]
*Lactobacillus acidophilus*	HIV infection	Gag	pTRK1035	(Constitutive)	Cell anchored	[82]
*Lactobacillus jensenii*	HIV infection	two-domain CD4 (2D CD4) proteins	pOSEL144	P_23_ (constitutive)	Secreted	[69]
*Lactobacillus casei*	Tetanus	Tetanus toxin fragment C (TTC)	pLP401-TTFC	P amylase (inducible)	Cell anchored	[72]
*Lactobacillus casei*	Tetanus	Tetanus toxin fragment C (TTC)	pLP501-TTFC	P_ldh_ (constitutive)	Cell anchored	[72]
*Lactobacillus plantarum*	Tetanus	Tetanus toxin fragment C (TTC)	pMEC160	P_ldh_ (constitutive)	Cell anchored	[70]
*Streptococcus gordonii*	Clostridium tetani infection	Tetanus toxin fragment C	pSMB158	(constitutive)	Cell anchored	[111]
*Lactobacillus acidophilus*	Helicobacter pylori infection	Adhesin Hp0410	pMG36e	–	Cytoplasmatic	[76]
*Lactococcus lactis*	Rotavirus infection	VP8	pNZ8048	P_nisA_ (inducible)	Secreted/cell anchored/cytoplasmatic	[112]
*Lactococcus lactis*	Malaria	MSP-1_19_	pL2-PSGT	–	Cytoplasmatic	[81]
*Lactococcus lactis*	Peanut allergy	Ara h 2	pNZ8148	P_nisA_ (inducible)	Secreted/cell anchored/cytoplasmatic	[93]
*Lactococcus lactis*	Dust mite allergy	Der p2	pNZ8148	P_nisA_ (inducible)	Secreted/cell anchored/cytoplasmatic	[94]
*Streptococcus gordonii*	Giardia lamblia infection	cyst wall protein 2 (CWP2)	pSMB104	(constitutive)	Cell anchored	[73]
*Lactobacillus zeae*	Streptococcus mutants infection	ScFv protein	pLP402-scFv	–	Cell anchored	[68]
*Lactococcus lactis*	Streptococcus pneumoniae infection	Pneumococcal surface protein A	pTREX1	P1 (pH dependent)	Cytoplasmatic	[113]
*Lactobacillus casei*	SARS-associated coronavirus infection	PgsA and spike protein	pHAT	P_HCE_ (constitutive)	Cell anchored	[114]
*Lactobacillus acidophilus*	Chiken anemia virus	VP1	pETacmA1	–	Cell anchored	[71]
*Lactococcus lactis*	Avian influenza virus	hemagglutinin 1 (HA1)	pMG36e	–	Cytoplasmatic	[91]
*Lactococcus lactis*	Leishmania major infection	LACK LACK + IL12	pDL-PnisA	P_nisA_ (inducible)	Secreted/cell anchored/cytoplasmatic	[88]
*Lactobacillus casei*	Pancreatic necrosis virus (IPNV)	VP2/VP3	pG1/pG2	Pxylose (inducible)	Secreted	[89, 90]
*Lactococcus lactis*	Body weight control	Leptin	pSEC:lep	P_nisA_ (Inducible)	–	[97]
*Saccharomyces cerevisiae*	Hypercalcemia	Salmon calcitonin	pAGA2-sCT	P_GAL1_ (inducible)	Cell anchored	[115]

Although LAB include microorganisms from different genus such as *Leuconostoc*, *Lactococcus*, *Lactobacillus*, *Pedicoccus* and *Streptococcus*, *Lactococcus lactis* has been the most widely used considering cloning and production of recombinant proteins [11]. *L. lactis* has been deeply characterized, being the first one whose genome was fully sequenced. In addition, it is an expression system easy to manipulate with many cloning and expression systems available. The most widely used protein expression system for *L. lactis* is the NICE (Nisin Controlled Expression) system, based on the control of a strong nisin inducible promoter (P_nisA_), which has several advantages. The expression of the gene of interest is tightly regulated and high expression levels are achieved using a food-grade molecule (nisin) as inducer. [12]. Although several proteins with biotechnological or biomedical interest produced in *L. lactis* using inducible plasmids have been proved in both experimental models and clinical trials [13], a prior induction of protein production have been required in these cases before the administration of the recombinant bacteria. For that reason, other inducible promoters that do not require the addition of any external inducer have been developed not only for *L. lactis*, but also for other LAB such as *Lactobacillus paracasei*. These promoters are directly induced in situ, for example once bacteria suffer environmental stresses such as heat-stress (body temperature is some degrees higher than bacteria optimal growth temperature) [14] or acid-stress (because of the stomach fluids) [15], enabling the recombinant production of the protein of interest without the need for adding an external inducer. In this regard and considering that *L. paracasei* respond to stress by synthesising chaperones such as groESL [14], an Stress-Inducible Controlled Expression (SICE) system based on the groESL operon promoter has also been described [15]. The development of promoters that do not depend on the addition of external inducers have allowed to take an important qualitative leap towards the use of LAB as protein delivery vectors. In this context and aiming to take another step forward, constitutive promoters are also being extensively studied. These constitutive promoters make possible to get a maintained expression of the protein of interest over time without the need of any type of inducer. Currently it has been widely explored, being possible to find an important number of examples that have already been tested for protein delivery purposes specially with *L. lactis*, but also with *L. paracasei*, *Lactobacillus casei*, *Lactobacillus plantarum*, *Bifidobacterium breve* and *Streptococcus gordonii* (Table 1).

**Fig. 1:**
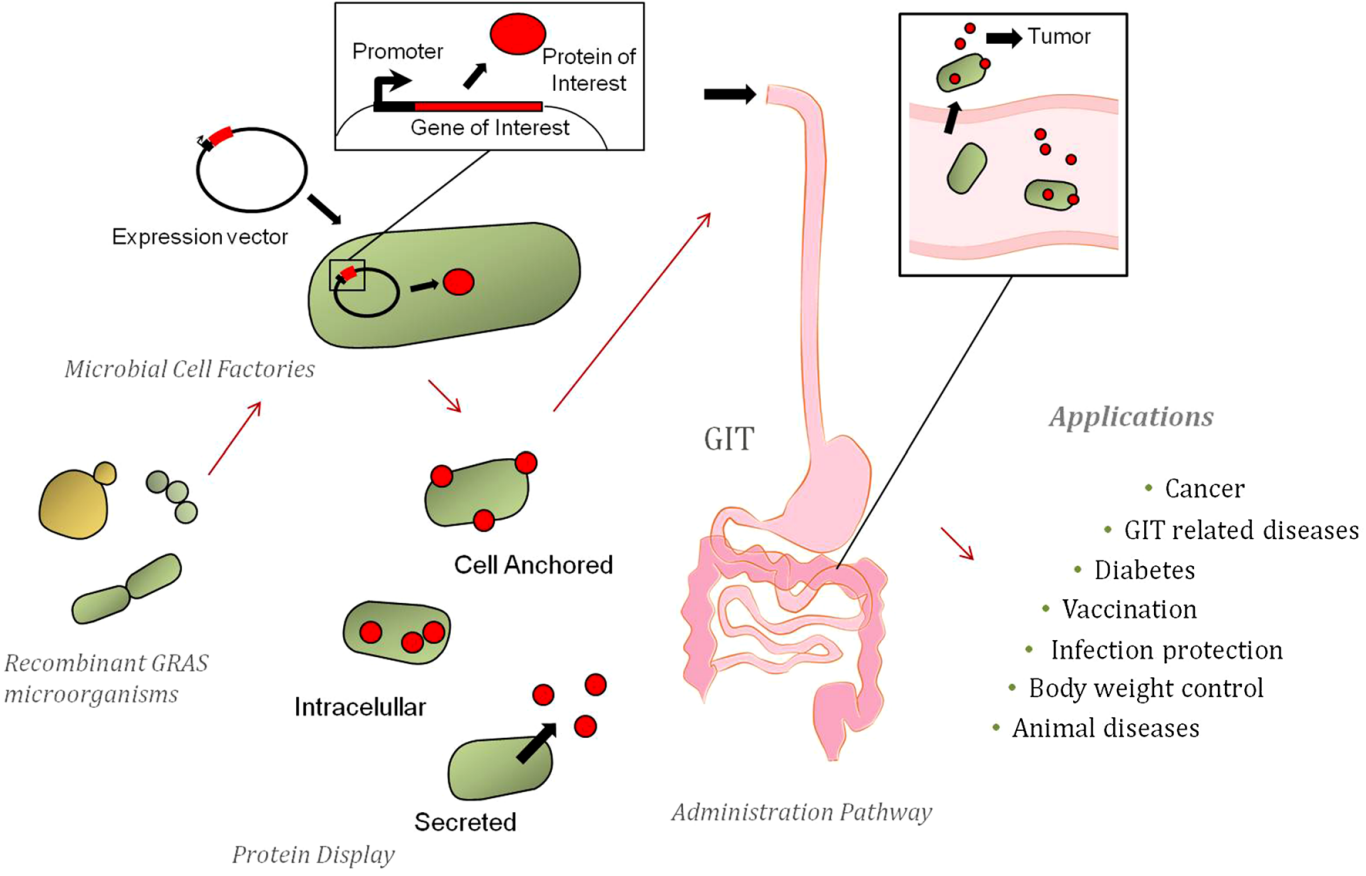
Schematic representation of the use of LAB for biomedical applications using the oral administration pathway

Thus, the use of food grade microorganisms as recombinant protein cell factories [9] and delivery platforms at the same time, is a promising approach [6, 11]. Briefly, the administration of such microorganisms would also suppose a significant decrease in the production cost of the drugs as being live organisms, these live vectors would be able to autonomously synthesize and deliver the prophylactic or therapeutic protein of interest. Moreover, it is possible to simultaneously produce different proteins in the same bacteria [16]. Altogether has turned them into an attractive alternative not only to intravenous administration of naked recombinant proteins, but also to other classical delivery systems for mucosal targeting, such as attenuated pathogens, liposomes and microparticles [10]. Thus, here, is intended to provide an overview of the use of genetically modified food grade organisms engineered as attractive vehicles for delivering functional proteins to mucosal tissues for the treatment of a wide range of pathologies such as GIT related pathologies as well as some types of cancer and viral infections, among others (Fig. 1).

## Review

### Autoimmune diseases

Effective therapeutic approaches for autoimmune diseases like GIT related diseases and diabetes are urgently needed and the development of oral formulation is an imperative need. Oral administration is the most preferred route since it is well accepted by patients, becoming a promising alternative for drug delivery of such autoimmune diseases.

#### GIT related diseases: Crohn’s disease and ulcerative colitis

Inflammatory bowel disease (IBD) is an idiopathic disorder consisting in the inflammation of the GIT. It is believed that this abnormal condition is due to an uncontrolled immune response against the gut microflora [17, 18]. Although the underlying cause is still unclear [19, 20], it is known that environmental and genetic factors have an important role in these complex diseases. Crohn’s disease and ulcerative colitis are included in this general IBD definition and the patients that suffer these chronic diseases usually require lifelong and costly treatments with sever side-effects. Moreover, in many cases, therapeutic agents used fail, and despite medical treatment, surgery is needed. In this sense, the use of recombinant microorganisms,  that fulfill the QPS standards, overexpressing any molecule able to alleviate inflammation could be an attractive and alternative treatment, since their safe profile and administration pathway would allow an easy incorporation of the treatment to the patient’s routine improving their comfort. Some strategies have been proposed using proteins such as low-calcium V antigen (LcrV) [21], anti-TNFα nanobodies [22], trefoil factors (TFF) [23], catalase [24, 25] and IL-10 [26] using *L. lactis* as microbial delivery vector (Table 1). All these proteins have been successfully produced by the *L. lactis* platform ameliorating, upon their oral administration, the inflammatory response of IBD animal models. Noteworthy, the success of these approaches is related to the delivery of the therapeutic agent at the mucosa level. A clear example is provided by the orally administered *L. lactis* secreting anti-TNFα nanobodies [22]. This nanobody secretion platform has an efficacy similar to that observed with the established therapy (Infliximab, Remicade), based on the intravenous infusion of anti-TNFα [22, 27–30]. However, contrary to what occurs by the systemic infusion, the oral administration of *L. lactis* strains secreting anti-TNFα is cost effective and lacks adverse effects [22].

LAB with potential in IBD treatment share the objective of reducing gut inflammation. Nevertheless, depending on the recombinant protein to be delivered the affected pathway differs. Thus, anti-TNFα nanobodies would reduce inflammation by neutralizing the action of the pro-inflammatory cytokine TNFα. Expression of IL-10, which is a regulatory cytokine, would decrease inflammation thanks to its antinflammatory activity [26], while the use of enzymes such as catalase would act on the inflammatory response derived from the presence of reactive oxygen species (ROS) [24, 25].

The approach that probably has been more extensively studied is the delivery of IL-10 produced in *L. lactis*. In fact, *L. lactis* secreting IL-10 has been submitted to clinical trials for the treatment of Crohn disease (ClinicalTrials.gov Identifier: NCT00729872) [23, 26, 31–34]. Nevertheless, and despite the very promising results observed in mice, the clinical trial revealed the mentioned approach inefficient (ClinicalTrials.gov Identifier: NCT00729872). This failure is thought as a consequence of a low final concentration of IL-10 in the GIT. In this regard, it has been recently proposed using IL-27, a pleiotropic cytokine, in order to get a broader response due to its immunosuppressive role as well as the capacity to induce IL-10 expression [35].

Besides, elafin, an endogenous protease inhibitor, has also been orally administered using *L. lactis* and *L. casei* as delivery vectors, observing a restoration of colon homeostasis in mice [11, 36, 37]. Elastin, which is diminished in patients with IBD, has a pleiotropic and anti-inflammatory role in healthy human gut [11, 36, 37]. Recently, two other anti-inflammatory molecules named secretory leukocyte protease inhibitor (SLPI) and the enzyme 15-lipoxygenase-1 (15-LOX-1) secreted by *L. lactis* have shown the ability to notably reduce the intestinal inflammation in mice [37, 38].

Other models used for the local display in the mice gut of therapeutic protein are *Lactobacillus gasseri* and *L. casei* both expressing superoxide dismutase (SOD) [39, 40]. As it happens with catalase, SOD action neutralizes ROS species and their derived inflammatory effect.

#### Diabetes

Some articles have also been published exploring the potential of food-grade bacteria for the treatment of diabetes (Table 1). In this context, Ng and collaborators proved that *L. lactis* is able to secrete an insulin analog in vitro [41], promoting the expected biological effect on target adipocytes. Some years later, Agarwal et al. have described a successful in vivo assay with rats based on the oral delivery of glucagon like peptide-1 (GLP-1) using again *L. lactis* as delivery platform [42]. Briefly, GLP-1 has emerged as a promising therapeutic peptide for type 2 diabetes treatment, being a compound that is synthesized by the GIT for the maintenance of glucose homeostasis. Up to now, GLP-1 has been administered through injection, being necessary one administration at least once a week. Interestingly, Agarwal et al. have observed that once recombinant *L. lactis* secreting GLP-1 is orally administered in rats, a reduction in blood glucose levels and an important increase in insulin take place [42].

Nowadays, it is widely accepted that current treatments based on insulin replacement for type 2 diabetes have important weak aspects [42]. On the one hand, the autoimmune response that impairs β-cells in pancreas is not inhibited [42]. On the other hand, insulin injection cannot prevent important associated complications to diabetes. Therefore, different therapeutic approaches based on immunotherapies are also being explored, being the use of antigen-based immunotherapies the most promising for this autoimmune disease. A 24 amino-acid peptide derived from human HSP60 has demonstrated to be a convenient alternative for the modulation of the immunological attack on β-cells in mouse [43]. This peptide was successfully orally administered using recombinant *L. lactis* as delivery carrier, having a clear effect on the improvement of glucose tolerance and in the reduction of insulinitis and hyperglycemia [43]. In addition, another study has been recently published describing the administration of a *L. lactis* strain delivering antigens such as pro-insulin or glutamic acid decarboxylase in combination with IL-10 and anti-CD3 as an appealing method to improve the induction of antigen-specific tolerance for the treatment of the type 1 diabetes [23, 44, 45]. Another study has been recently followed the oral administration of *L. lactis* secreting two major auto-antigens of type 1 diabetes, named glutamic acid decarboxylase (GAD65) and tyrosine phosphatase-like protein ICA512 (IA-2) in mouse models [23, 46]. In this study, modified versions of GAD65 and IA-2 have been successfully used in combination with human IL-10 cytokine [23, 46].

### Cancer

Cancer has a huge relevance in human health due to its growing incidence in developed countries. The strategies for effective cancer treatment under study are countless and the use of LAB has recently appeared in this field.

Cancer development and progression have been broadly related with chronic inflammation processes produced by external factors such as infection, radiation, unbalanced diet, obesity, tobacco or the exposure to other environmental pollutants [47]. Thus, in principle, any strategy aimed to treat chronic inflammation could produce also a positive outcome in cancer occurrence. In this regard, the use of LAB organisms for colorectal cancer prevention has been explored using mainly colorectal cancer murine models [48]. Some examples of the strains used with this purpose are *Bifidobacterium lactis* [49]*, L. casei strain Shirota* [50]*, B. longum BB536* [51]*, Lactobacillus acidophilus Delvo Pro LA*-*1* [52]*, Lactobacillus rhamnosus GG* [53] *or Propionibacterium freudenreichii* [54] but many others rendered similar results, showing a significant decrease in cancer development. It is important to note although the significant number of LAB showing promise in in vitro and in animal models that no conclusive studies have been carried out in humans.

On the other hand, interesting studies regarding biodistribution of the microbial vectors in mice models illustrates the capacity of some food grade species to reach solid tumors, where they are able to accumulate and proliferate after intravenous administration [55, 56]. This behavior has been related with the hypoxic environment exhibited in the tumors. In such atmosphere anaerobic bacteria selectively grow [55, 56]. This capacity has been exploited in *B. longum* to propose an anti-breast cancer strategy based on the recombinant production of cytosine deaminase in the solid tumor after the intravenous administration of the microbial vector. The enzyme combined with the administration of the 5-fluorocytosine (5FC) would result in a locally high concentration of the reaction product, 5-fluorouracyl (5FU) [57]. Another example using *Bifidobacterium adolescentis* expressing a recombinant endostatin showed how this safe vector was able to selectively inhibit angiogenesis and tumor growth in tumor mice models after its intravenous administration [58]. These studies reinforce the capability of these microorganisms’ genera, classified according to QPS standards, as potential drug delivery systems for cancer treatment. The protein secreted by the live vector will be more stable than those naked soluble proteins intravenously administered. However, as previously mentioned, the intravenous injection of LAB have important adverse effects. Thus, the delivery of such live vectors at mucosal level would be much more appropriate. In this context, the potential use as orally administered drug delivery vector with a natural selectivity for solid tumors has also been explored (Table 1). Recombinant *B. breve* orally administered in mice are able to effectively translocate the GIT and colonize solid tumors at the same levels than intravenously administered ones [59]. Interestingly, the crossing of the GIT by the recombinant *B. breve,* involving an increased permeability of the GIT epithelia didn’t promote the crossing of potentially pathogenic bacteria present in the regular gut flora [59].

Besides, LAB can also be orally administered taking advantage of their natural niche in the body to develop prophylactic strategies against colon cancer without the need of the GIT translocation. In this sense a recombinant *L. lactis* producing catalase has shown a protective effect in chemically induced colon cancer in mice models [60]. Tumor cells are characterized by an increased production of ROS such as hydrogen peroxide (H_2_O_2_), that actively participate in enhancing tumour invasion and proliferation. Thus, the administration of *L. lactis* producing catalase, an enzyme with an antioxidant activity, decreases H_2_O_2_ levels and, consequently, reduces colonic damage and inflammation [60]. Interestingly, since the oxidative stress associated to an increase of ROS levels is also characteristic of gastrointestinal pathologies, the approach developed in this study can be used also as a therapy for the treatment of IBD. Recently, some articles have been published using the administration of *L. lactis* expressing human papillomavirus E7 oncoprotein (HPV-16 E7) for the treatment of cervical cancer. In one of these articles, E7 protein has been produced by a secretion SICE plasmid and administrated to mice with tumours. Results show that administration of recombinant bacteria provokes a slightly diminution of tumour volume and an antigen-specific immune response [15]. In other studies these food-grade bacteria have been administered in mice via intranasal expressing HPV-16 E7 anchored to its surface [61, 62]. In one case, E7 protein has been combined with calreticulin-E7 administration inducing >80 % of tumour suppression in mice [61]. In a second approach, recombinant lactococci have been tested for the simultaneous delivery of E7 protein and murine interleukin-12 (IL-12) DNA [62], observing that this new strategy combining the delivery of both the therapeutic molecules and antigens has a high potential. Finally, E7 protein effect has also been investigated using *L. casei* as mucosal delivery vector in mice [63].

### Infectious diseases

Historically vaccines have been based on attenuated pathogenic microorganisms [64, 65]. However, this approach has three important drawbacks: (a) difficulties on the construction of stable attenuated mutants; (b) presence of residual virulence in attenuated pathogens; (c) risk of genetic reassortment between the vaccine strain and the wild type. Besides, although pure antigens have also been used for vaccination purposes, these molecules have a low or non-existing immune response, especially because their rapid degradation and their poor adsorption in vivo [66]. Thus, aiming to find an alternative strategy, non-pathogenic LAB have also been explored as mucosal vaccines. Many approaches have been proposed in order to produce and present different antigens [23, 67]. Most of them have been developed in *L. lactis,* but also there are works using other LAB such as *Lactobacillus zeae* [68], *Lactobacillus jensenii* [69], *L. plantarum* [70], *L. acidophilus* [71], *L. casei* [72], *Streptococcus gordinii* [73], *and Bacillus subtilis* [74] (Table 1). It should be noted that several *Lactobacillus* species have been exploited in this field, being some of them able to attach and colonise the gastrointestinal mucosa, being acid-resistant and biletolerant [75]. Besides, the presence of these bacteria may naturally inhibit the pathogenic colonization of pathogenic microorganisms such as *Helicobacter pylori* [76]. However, although *Lactobacillus* seems to be one of the best candidates for immunization purposes, protein expression levels achieved are still lower than those obtained with *L. Lactis* [66, 77]. Since low protein yields cannot induce an immune response strong enough to trigger protection against infective agents, an optimal antigen presentation is required. That is possible with a sudden high concentration of the antigen as such obtained with inducible approaches [67]. Nevertheless, given that it is common to find either insolubility or toxicity of some recombinant proteins during overexpression, constitutive plasmids can be a good alternative [78]. The use of constitutive plasmids is a much safer approach, being not necessary to add any external inducer to get the desired amount of protein. The use of constitutive plasmids is exemplified by the expression of SspA and SspB antigens from *S. gordonii*, a major colonizer of oral hard and soft tissues, on the cell surface of *L. lactis* using P1 promoter. Both antigens were successfully expressed and anchored in the cell wall and their in vitro ability to adhere to *S. gordonii* surface was proven [79]. Another example is the *slpA* constitutive promoter based on S-layer protein. The very strong expression signal of S-layer has been proven for the secretion of β-lactamase using *L. lactis*, *L. brevis*, *L. plantarum*, *L. gasseri* and *L. casei* [80] and of Merozoite surface protein 1 (MSP1) from *Plasmodium falciparum* using *L. lactis* [81]. Interestingly, this last study proves the potential of recombinant *L. lactis* as an effective oral vaccination alternative against malaria [81]. In vivo studies with MSP1 antigen show its capacity to confer protection to the vaccinated animals [81]. Furthermore, the combination of the antigen with adjuvant molecules have been studied also aiming to achieve an increased efficiency of these vaccines. An example is the coexpression of HIV-1 with the flagellin (FliC) of *Salmonella enterica* in *L. acidophilus*. The results show that Gag (antigen against HIV-1) and FliC coexpression promotes Gag-specific IgA-producing cells at the local mucosa [82]. Another study has been reported aiming to develop a vaccine against the chicken anemia virus (CAV) [71]. VP1 antigen produced in *E. coli* was fused to the binding domain of AcmA, the major autolysin of *L. lactis* cell wall, at N-terminal aiming to enhance the *Lactobacillus* immunization ability. In this case, the authors observed that the fusion protein remains on the cell surface at least 5 days and that the oral administration induced a moderate immune response in chicken [4, 71].

*Bifidobacterium* is another appealing food-grade vector. It is abundant in human gut, well recognised as probiotic and with the ability to activate Th1 cell-mediated immune responses without antigen presentation [83]. Because of its biletolerance, a regulated promoter based on upstream sequence of *bet A* (a bile-inducible transporter gene) has been recently developed in order to control gene expression specifically in the intestinal tract [84]. However, it should be noted that *Bifidobacterium* has a strict anaerobic metabolism making harder the experimentation with it.

A part from IBD, diabetes and cancer, other diseases have been targeted using food grade bacteria. Among them, interesting examples can be found in the control of microbial infections (Table 1). Regarding microbial infections neutralizing antibody fragments expressed in *L. paracasei* and *L. acidphilus* have been shown able to provide protection against *Bacillus anthracis* [85, 86] and *L. paracasei* against rotavirus [87] in mice. These studies open possibilities of generating alternative *Lactobacilli* producing antibodies against other infectious diseases affecting the GIT.

Besides, it is important to note that LAB and other organisms classified as QPS by EFSA are useful mucosal delivery vectors to treat not only human diseases but also animal diseases. Just as an example, LAB have been used to combat Leishmaniasis [88]. Moreover, *Lactobacilli* have also been used to design live vaccines to combat a wide range of diseases such as pancreatic necrosis virus (IPNV, a pathogen that infects wild and cultured salmonids) [89, 90], a highly pathogenic avian influenza (HPAI) [91], and porcine epidemic diarrhea virus (PEDV) [92].

### Allergic and other diseases

Some studies have combined the bacterial effect with an expressed antigen either for food hypersensitivity or aero-allergens. The administration of *L. lactis* displaying the recombinant allergen intracellularly, in the extracellular space or cell wall-anchored show its capacity to modulate the Th2-based specific antibody responses, in the case of the allergen Ara h 2 against peanut allergic [93] and the Der p2 allergen against the dust mite allergy [94]. In the second study, the authors also observed a diminution in the cellular infiltration and inflammatory response.

It has been demonstrated that obesity and gut microbiota composition strongly correlates [95, 96]. In addition, some LAB such as *Lactobacillus* and *Bifidobacterium* are able to improve obesity in both murine models and humans [96]. In this context, obesity has also been targeted in mice models by the delivery of recombinant leptin using LAB. Leptin is a hormone with a crucial role in body weight control. In this case, the capability of this protein to carry out its action was improved by the intranasal administration of engineered *L. lactis* secreting the hormone observing a significant loss in body weight as well as a reduction in food intake in the treated animals [97].

Another example is the administration of cytokines-secreting LAB as prophylaxis therapy, being the delivery of IL-10 for inflammatory bowel diseases [98] and of IL-12 for asthma [99] just a couple of examples [100].

Finally it is necessary to stress out that although most of the applications referred to in this revision are envisaged using LAB, also eukaryotic expression systems with a safe profile can be found in the literature. This possibility would result of interest in the cases of therapeutic proteins requiring complex post-translational modifications to became fully functional [101]. In this regard, prokaryotic cell factories would lack the machinery to perform the required processing of the recombinant protein [101]. A nice example is found in recombinant *Saccharomyces cerevisiae*. This yeast has been proposed as a vehicle to secrete proteins or peptides with a therapeutic effect in the gut [102]. In this sense, an orally recombinant *S. cerevisiae* displaying a salmon recombinant calcitonin on the yeast surface prompted a decrease in calcium levels in hypercalcemic rats after oral administration.

### Adverse side-effects of LAB

In the previous sections we have been focusing the attention on the positive effect of LAB as live vectors for protein delivery. However, it is important to stress that LAB are genetically modified organisms (GMOs). GMOs are widely accepted and well stablish in food industry. However, important regulatory concerns need to be addressed for its use as therapeutic vechicles. Specifically, LAB are based on expression systems carrying antibiotic resistance genes as a selection marker [103]. It has been described that these live vectors could transfer its antibiotic resistances to intestinal microbiota. Although this is a really rare event that has not been reported in this field, it is an important issue to be considered. The application of LAB as live vectors opens a broad and interesting field of possibilities, but regulatory measures have to be considered to ensure the safety of the used strains. Up to now, some alternative and innovative selection markers have already been developed, being some of them successfully tested and positively evaluated by several health authorities [23, 34, 103]. However, these alternative and safe selection markers need to be further explored to finally ensure the real possibility of using such strains for the problems listed above.

Besides, it should be noted that some adverse effects of LAB have been reported [104–106]. This indicates that, despite the positive therapeutic effects of these microorganisms and the low number of adverse effects registered, they are not completely safe. This information should be contextualized, since the adverse effects were observed in high risk groups such as critically ill and/or immune-compromised patients, critically sick infants, and postoperative and hospitalized patients [104]. Sepsis, fungemia and GI ischemia are the main harmful effects of LAB described [104]. Briefly, in this vulnerable populations LAB could interfere with the microflora giving rise to opportunistic infections and finally to bacterimia, fungimia or other medical complications. In addition, there are strong evidences proving that LAB, when used as probiotics, have anti-inflammatory effects. However, many reports also describe pro-inflammatory effects caused by such group of bacteria [107, 108]. This means that probiotic effect of LAB is strain dependent, being a factor to be considered for the choice of host strains for therapeutic applications.

In general terms, one can conclude that the safety of LAB is widely supported by the long tradition of use of such microbes. This safety record leads us to conclude that risk–benefit ratio in the prevention and treatment of multiple disease states is overall really high, being the studies reporting adverse effects scarce. Nonetheless, risks and benefits should be carefully considered in each situation, especially on those health-compromised patients. Besides, considering that adverse events are poorly documented, an accurate safety report including pathogenicity, infectivity, virulence and toxicity would help the scientific community taking decisions and more solid conclusions [105].

## Conclusions

The irruption of nanotechnology and other innovative approaches have allowed the development of alternatives to the classical medicine aiming to overcome its inherent limitations. In this regard, the exploitation of LAB as recombinant probiotics expressing any protein of interest has strongly burst them as a promising alternative for the treatment of a wide range of diseases. The mucosal, needle-free, administration of therapeutic molecules of interest gives an added value to LAB. Besides, it has been shown that the application of these recombinant probiotic bacteria via intranasal, oral or genital would have a dual effect: a direct effect designed for the treatment of a specific disease through the expression of a recombinant protein, combined with the indirect and general effect that some of these safe bacteria have in health. Additionally, the administration of such live delivery vectors is easier and relatively inexpensive compared to injectable treatments, being a large-scale production affordable [66]. Interestingly, up to now the use of LAB has been successfully tested for a wide range of medical applications, mainly using animal models, being the treatment of autoimmune diseases the most intensively investigated. These food-grade bacteria have also been proposed as excellent candidates for vaccination. There is still a lot to be done and a long way to reach the market, but all the articles published up to now let us suggest LAB as a promising delivery vector for a vast range of biomedical applications.

The next step will be the detailed study of all factors that could become an important bottleneck in the future implementation of LAB as effective live vectors delivering proteins of interest in situ. Important safety and regulatory issues still need to be addressed in depth, but some significant steps have already been done in this context. Small trials using auxotroph strains have been positively evaluated for the treatment of patients with Chron’s disease [26, 34]. Other aspects such as an extensive study of real cost and efficiency are still outstanding questions that need to be answered.
